# MPL Adjuvant Contains Competitive Antagonists of Human TLR4

**DOI:** 10.3389/fimmu.2020.577823

**Published:** 2020-10-16

**Authors:** Yi-Qi Wang, Hélène Bazin-Lee, Jay T. Evans, Carolyn R. Casella, Thomas C. Mitchell

**Affiliations:** ^1^School of Pharmaceutical Science, Zhejiang Chinese Medical University, Hangzhou, China; ^2^Center for Translational Medicine, University of Montana, Missoula, MT, United States; ^3^Department of Microbiology and Immunology, School of Medicine, University of Louisville, Louisville, KY, United States

**Keywords:** monophosphoryl lipid A (MPL), Toll-like receptor 4 (TLR4), partial agonist, vaccine adjuvant, reactogenicity, safety

## Abstract

Monophosphoryl lipid A (MPL^®^) is the first non-alum vaccine adjuvant to achieve widespread clinical and market acceptance, a remarkable achievement given that it is manufactured from a *Salmonella enterica* endotoxin. To understand how MPL^®^ successfully balanced the dual mandate of vaccine design—low reactogenicity with high efficacy—clinical- and research-grade MPL was evaluated in human and mouse cell systems. Stimulatory dose response curves revealed that most preparations of MPL are much more active in mouse than in human cell systems, and that the limited efficacy observed in human cells correlated with TLR4 inhibitory activity that resulted in a partial agonist profile. Further analysis of the major components of MPL^®^ adjuvant prepared synthetically identified two structural variants that functioned as competitive antagonists of human TLR4. A partial agonist profile could be recapitulated and manipulated by spiking synthetic agonists with synthetic antagonists to achieve a broad dose range over which TLR4 stimulation could be constrained below a desired threshold. This report thus identifies mixed agonist–antagonist activity as an additional mechanism by which MPL^®^ adjuvant is detoxified, relative to its parental LPS, to render it safe for use in prophylactic vaccines.

## Introduction

Monophosphoryl lipid A (MPL^®^) is the first vaccine adjuvant to achieve clinical and market success since the advent of aluminum salts in the early 20^th^ century. It was first authorized as a component of adjuvant system 4 [AS04 (1)], aluminum hydroxide semi-crystalline gels adsorbed hydrostatically with MPL, in the HBV vaccine Fendrix (2) for use in patients with renal insufficiency and then for broader use in the HPV vaccine Cervarix (3, 4). Formulations that lack alum completely, such as adjuvant system 1 [AS01 (5)] that contain MPL and QS-21 in liposomal complexes similarly achieved success as the adjuvant component of the varicella zoster vaccine Shingrix (6, 7). Because MPL^®^ is a highly purified derivative of the lipopolysaccharide (LPS) component of the cell-wall of *Salmonella enterica* its success as an adjuvant has been understood primarily in the context of its activity as an agonist of TLR4 that directly activates dendritic cells (8). TLR4-mediated activation of antigen-presenting and innate immune cells leads to mildly inflammatory conditions (9) that favor Th1 differentiation and production of Th1-associated humoral responses (10). MPL^®^ adjuvant therefore provides a counterbalance to the Th2-differentiating properties of alum (11) by harnessing the immunostimulatory power of TLR4 activation. The clinical success of MPL^®^ adjuvant in the context of prophylactic immunization is a benchmark achievement that bodes well for further use of TLR agonists as vaccine adjuvants, provided we can learn to deploy them safely.

Edgar Ribi’s goal in creating MPL^®^ adjuvant was to generate a compound that retained the tumoricidal properties of LPS while lacking its dangerous endotoxicity (12). In collaboration with Kuni Takayama an acid hydrolysis method was developed to convert the LPS of *Salmonella enterica* serovar Minnesota Re595 into a low toxicity, monophosphoryl lipid A (13–15). An additional base hydrolysis modification was added by Kent Myers and colleagues in order to remove one specific fatty acid and further decrease residual endotoxicity (10, 16). Clinical-grade MPL^®^ is manufactured exclusively by GlaxoSmithKline for use as an adjuvant in its vaccine portfolio whereas ‘generic’ forms of it, which will be designated as MPLA hereafter, are readily available from a variety of commercial suppliers. These research-grade MPLA preparations are also derived from the LPS of *Salmonella enterica* serovar Minnesota Re595 but are made without application of the base hydrolysis step and therefore differ from MPL^®^ in ways that make it harder to investigate which properties make it a successful adjuvant.

The complexity of the process used to manufacture MPL^®^ from LPS, needed to ensure it is both safety and functionality, generates multiple congeneric forms with anywhere from three to six acyl chains (10, 13, 14, 17–20). The hexa-acylated species is generally highlighted when structures of MPL^®^ are published (21), even though it is not the most abundant (17) and the penta-acylated species is fully functional as an adjuvant in mice (17). The focus on the hexa-acylated species was driven in part by a view of the hexa-acyl Lipid A of *Escherichia coli* as representing the optimal structure for activation of TLR4 (10), which has led to use of synthetic hexa-acylated structures corresponding to the *E. coli* chemotype (22, 23) or hexa-acylated monosaccharide mimetics in pre-clinical and clinical vaccine trials (24–26). Notably, tetra-acylated Lipid IVa is an agonist of mouse TLR4 but a competitive antagonist of human TLR4 (27), part of a broader theme that emerged in which human TLR4 is better able to discriminate amongst different LPS and Lipid A structures, relative to mouse (28). Hence, the multiple, congeneric Lipid A species in MPL^®^ adjuvant may cause its activity in pre-clinical animal models, such as mice, to be very different from that observed in human cell systems.

In this study we sought to evaluate differences in stimulation of human and mouse immune cells by first using research-grade MPLA preparations and then turning to vaccine suspensions containing clinical-grade MPL^®^ adjuvant in the form of AS01 and AS04 (Shingrix and Cervarix, respectively). We also obtained and tested synthetic preparations of the major components of MPL^®^ adjuvant and report for the first time the presence of competitive antagonists of human TLR4, which may impose an upper limit on the extent to which the MPL^®^ mixture can activate inflammatory side effects.

## Materials and Methods

### TLR4 Reagents

Research-grade MPLA (4′-monophosphoryl lipid A) prepared from *Salmonella enterica* serovar Minnesota Re595 LPS by acid hydrolysis was purchased from Avanti Polar Lipids (A-MPLA), Enzo Life Sciences (cat.no. ALX-581-202, E-MPLA), Invivogen (I-MPLA) and Sigma-Aldrich (S-MPLA) all of which are heterogeneous mixtures estimated to average ~MW 1,700 g/mol. Clinical-grade MPL adjuvant^®^ manufactured from *Salmonella minnesota* Re595 LPS by acid and base hydrolysis (resulting in a mixture of 3-*O*-desacyl-4′-monophosphoryl lipid A congeners with three to six acyl chains) was tested in the form of Cervarix vaccine, an aluminum hydroxide-adsorbed suspension containing 100 μg/ml MPL adjuvant (AS04) plus recombinant HPV virus-like particles, and the adjuvant vial from Shingrix vaccine (*i.e.*, without mixing in lyophilized glycoprotein E from the separate antigen vial), a dioleoyl phosphatidylcholine (DOPC) liposomal formulation containing 100 μg/ml MPL^®^ adjuvant, the immunostimulatory saponin QS-21, and cholesterol (AS01_B_).

Tandem mass spectrometry was performed through the Analytical Services Division of Avanti Polar Lipids, Inc. (Alabaster, AL) to evaluate the composition of research-grade MPLA preparations. Briefly, samples of each preparation, 100 μg, were dissolved in 1 ml of 70:28: methanol:dichloromethane:water and assayed by LC/MS/MS to determine the relative abundance of MPLA molecular species with four to seven acyl chains using the same Q1/Q3 precursor ion/product ion analytic method Avanti employs in analysis of its *S. minnesota* Re595 MPLA product (A-MPLA).

Reference TLR4 agonists included LPS from *Salmonella enterica* serovar Minnesota Re595 (MW ~2,300 g/mol, Enzo Life Sciences cat. no. ALX-581-008); synthetic Lipid A (MW 1,797.2 g/mol, Peptides International cat.no. CLP-24005-s) with 1,4′ phosphates and six acyl chains in a 2:2:1:1 configuration corresponding to the *E. coli* chemotype (aka LA-15-PP or compound 506); phosphorylated hexa-acyl disaccharide (PHAD, MW 1,763.5 g/mol, Avanti Polar Lipids cat. no. 699800P), a synthetic MPLA with a 4′ phosphate and six acyl chains corresponding to the *E. coli* chemotype; and 3D(6-acyl) PHAD (3D-6A-PHAD, MW 1,747.5 g/mol, Avanti Polar Lipids cat. no. 699855P) a synthetic variant of PHAD with acyl chains in a 2:2:0:2 configuration corresponding to the hexa-acylated component of clinical-grade MPL adjuvant^®^. Acyl chains in the PHAD synthetics are uniformly C14 in length, whereas the Lipid A synthetic has one secondary acyl chain that is C12.

Individual components of MPL adjuvant^®^ were synthesized as described previously (18, 19) as 3-*O*-desacyl-4′-monophosphoryl lipid A congeners with varying numbers of acyl chains corresponding to the major components found in clinical-grade MPL adjuvant^®^: hexa-acylated (hereafter referred to as ML6, 2:2:0:2 acyl chain configuration), penta-acylated (ML5A and ML5B, 2:2:0:1 and 1:2:0:2 acyl chain configurations, respectively), tetra-acylated (ML4A and ML4B, 1:2:0:1 and 0:2:0:2 acyl chain configurations, respectively), and tri-acylated (ML3, 0:2:0:1 acyl chain configuration) monophosphoryl lipid A. The MPL^®^ adjuvant synthetics reflect differences in acyl chain lengths of *S. enterica* Minnesota LPS in which the 2′-secondary acyl chain is C12, and the 2-secondary acyl chain is C16 in length.

LPS, Lipid A, and monophosphoryl Lipid A reagents other than Cervarix and the adjuvant vial of Shingrix were suspended in endotoxin-free water containing 2% glycerol by sonication at 80 kHz in an Avanti Sonicator (model G112SP1TB) until clear.

### Cells

Human and mouse HEK-Blue reporter cells express human or mouse CD14, MD2, and TLR4 and secreted embryonic alkaline phosphatase (SEAP) under control of a promoter with binding sites for NFκB and AP-1 transcription factors (InvivoGen cat. nos. hkb-htlr4 and hkb-mtlr4). Cells were cultured as directed by the supplier, with the exception that Glutamax (ThermoFisher cat. no. 10569010) was used in place of L-glutamine, and TrypLE Express (ThermoFisher cat. no. 12605010) was used to release adherent cells for each passage. BALB/c mouse RAW264.7 and human THP-1 cells (ATCC^®^ cat. nos. TIB-71 and TIB-202) were cultured in DMEM (4.5 g/L D-Glucose with 2 mM L-glutamine, 1 mM sodium pyruvate, 100 U/ml penicillin and 100 μg/ml streptomycin) supplemented with 10% heat-inactivated FBS. THP-1 cells were exposed to phorbol-12-myristate-13-acetate (PMA, InvivoGen cat no. tlrl-pma), 5 ng/ml, for 48 h prior to treatment with TLR4 agonists. Bone marrow-derived dendritic cells (BMDCs) were generated using C57BL/6 mouse femurs following the exact method published by Lutz et al. (29) except that 5 rather than 20 ng/ml recombinant GM-CSF was used to differentiate the DC. Human peripheral blood monocytes (PBMCs) were prepared from venous blood of healthy volunteers that had been collected using sodium citrate as anti-coagulant, 20 ml of which was mixed with 15 ml calcium- and magnesium-free Hank’s Balanced Saline Solution (HBSS) and then laid over 6 ml Histopaque^®^-1077 (1.077 g/ml, Sigma-Aldrich cat no. 10771) for density gradient centrifugation. The buffy coat layer was collected by pipette, washed once in HBSS, once in serum-free RPMI-1640, and then the PBMCs were resuspended in RPMI-1640 supplemented with 100 U/ml penicillin, 100 g/ml streptomycin, 1 mM Na-Pyruvate, 2 mM L-glutamine, 50 μM *β*ME and 10% heat-inactivated, male AB human serum for culture.

### Assays

SEAP assays were performed to evaluate the extent to which TLR4 signaling was activated in HEK-Blue reporter cells expressing mouse or human receptors (CD14, MD2 and TLR4). Reporter cells were harvested when 70–80% confluent and plated in 96-well, flat-bottom microtiter plates either 1 h, one day or two days (5 × 10^4^, 4 × 10^4^ or 2 × 10^4^ cells/well, respectively) before addition of TLR4 agonists for 18–24 h. Culture supernatants were transferred to U-bottom microtiter plates, centrifuged and transferred again to microtiter plates to ensure reporter cells were not present. The culture supernatants were added to QUANTI-BLUE substrate following instructions provided by the manufacturer (InvivoGen cat. nos. rep-qbl or rep-qbs) and incubated at 37°C in a tissue culture incubator. At successive intervals beginning 10 min after mixing the supernatants and substrate, the plates were placed in an Emax plate reader (Molecular Devices LLC) and optical density was measured at visible light wavelength 650 nM (OD_650_). The shortest incubations at which dose response curve fits produced maximal R^2^ ‘goodness of fit’ values, as determined by GraphPad Prism software, were used for analysis.

ELISA was performed to measure TNFα secretion by cell-lines and primary cells exposed to TLR4 reagents as a measure of receptor activation. Human PBMC, 5 × 10^5^/well, or THP-1 cells, 5 × 10^4^/well, and mouse BMDC or RAW264.7, 10^5^/well, were plated in microtiter plates to which TLR4 reagents were added for 18–24 h. Culture supernatants were transferred to U-bottom microtiter plates, centrifuged, transferred again to fresh microtiter plates to ensure cells were no longer present and then frozen until needed. Human or mouse TNα was quantified using ELISA kits as directed by the kit supplier (eBioscience cat. nos. 88-7324-77 and 88-7324-88, respectively) using an Emax plate reader and SoftMax Pro analytical software (Molecular Devices, LLC). Culture supernatants from replicate freezing were diluted as needed to ensure unknown sample values were within the range observed in the standard curves.

### Pharmacological Dose Response Curves

Pharmacological profiles of the TLR4 reagents were generated by performing dose response curves in one of four formats. **1**) Agonist potency and efficacy (EC50 and height of dose plateau, respectively) were measured by diluting TLR4 reagents successively in complete culture medium [DMEM or RPMI supplemented with fetal bovine serum (FBS) or RPMI supplemented with human serum, depending on cell-type]. Half-log step dilutions were performed in 96-well, U-bottom microtiter plates using a multichannel pipette to transfer and mix the suspensions, with fresh tips used for each dilution step. One-tenth (20 μl) volumes from wells of the dilution plate were then transferred to wells containing cells that had been plated in nine-tenth volumes (180 μl) of culture medium. **2**) Mixed agonist–antagonist potency and efficacy were measured by mixing a TLR4 agonist with a candidate antagonist at a fixed ratio and then performing half-log step dilutions before treating HEK reporter cells. Analysis of values obtained from SEAP assays of culture supernatants was then performed as described for preparations containing agonist alone, above. **3**) Competitive *vs* allosteric or non-competitive antagonist type was evaluated by diluting a reference agonist within a fixed concentration of a candidate inhibitor in half-log steps, treating HEK reporter cells, performing SEAP assays and then analyzing the data as described for agonist assays, above. Competitive antagonists are characterized by values that reach the same dose plateau as for a reference, full agonist, while shifting potency to higher EC50 values. Non-competitive antagonists are characterized by values that do not reach the same dose plateau as a full agonist, indicating that overabundance alone of the antagonist cannot compete with the agonist for binding to activation sites. **4**) Inhibitory potency (IC50) was measured by diluting antagonists within a fixed concentration of a reference agonist, treating HEK reporter cells and performing SEAP assays as described.

### Statistical Analysis

All statistical tests were performed using GraphPad Prism version 8.4.3 for Windows, GraphPad Software, San Diego, California USA, www.graphpad.com. Assay results were graphed as means ± standard deviations (SD) pooled from multiple independent experiments and replicate treatment groups as specified in each figure legend. Dose stimulation and IC50 assay results were additionally fit by regression curves using the Prism log(agonist) *vs.* response—variable slope (four parameters) and the log(inhibitor) *vs.* response—variable slope (four parameters) equations, respectively. Each curve fit is depicted as a solid line with shaded boundaries representing 99% confidence intervals, or the space that would describe the true value of the population mean in 99% of experimental repetitions. These 99% confidence intervals are equivalent to p values ≤ 0.01 for curves whose shaded boundaries do not overlap. In **Figure S1** the relationship between abundance of a given MPLA component and the top dose plateau (apparent efficacy) was evaluated using Prism multiple variable tables to calculate non-parametric Spearman coefficients with P values computed from two-tailed tests of significance of the correlations.

## Results

### Heterogeneity of Research-Grade MPLA

**Figure 1:**
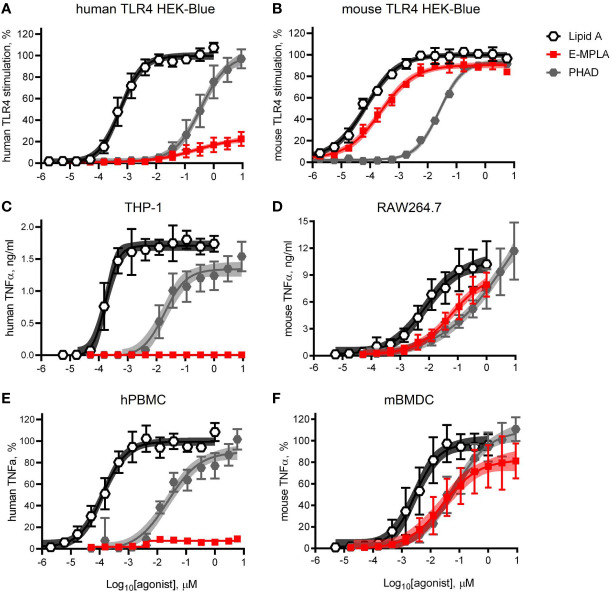
Partial agonism of TLR4 by research-grade MPL adjuvant is species-specific. HEK-Blue reporter cells, monocytic cell-lines, and primary cells of mouse and human origin were exposed to research-grade E-MPLA to compare dose response profiles. Dose curves were performed as half-log step dilutions from a peak concentration of 10 μM with exposure of cells for 18–24 h to synthetic *E. coli* chemotype Lipid A, its monophosphoryl counterpart PHAD, or MPLA from Enzo Life Sciences (E-MPLA). **(A, B)** show normalized responses of HEK-Blue reporter cells engineered to express human TLR4 or mouse TLR4 respectively; 100% = top dose plateau of the full agonist Lipid A. **(C, D)** show TNFα production by human monocytic THP-1 or mouse RAW264.7 cells, respectively. **(E, F)** show normalized TNFα production by human PBMC or mouse BMDC, respectively; 100% = top dose plateau of the full agonist Lipid A Average values ± SD combined from three **(A, B, D, F)** or two **(C, E)** independent experiments, each performed in triplicate, are shown along with shaded regions that indicate the 99% confidence intervals within which the true population means should occur 99% of the time. In each paired comparison research-grade MPLA-E scored as a partial agonist of human TLR4 not mouse TLR4, relative to the full agonists Lipid A (full/potent) and PHAD (full/weak).

Research-grade MPLA is often used in pre-clinical mouse studies as a surrogate for MPL^®^ adjuvant. We decided to compare the relative activities of one commercial preparation of MPLA, denoted here as E-MPLA, that we have used in previous mouse studies in mouse *versus* human systems. For these first experiments, we deployed matched pairs of cell types to evaluate species-specific differences: human or mouse TLR4 HEK-Blue reporter cells, human THP-1 or mouse RAW264.7 cell-lines, and primary PBMC from healthy blood donors or bone marrow-derived dendritic cells from mice (**Figures 1–A**). In each comparison synthetic Lipid A was the most potent of the agonists tested and reached dose curve plateaus that provided a benchmark for the upper limit of TLR4 signaling capacity for each cell type. The monophosphorylated counterpart to Lipid A, PHAD, was consistently less potent than Lipid A but had the same potential to saturate TLR4 signaling capacity at high doses, whether human or mouse. Monophosphoryl PHAD is thus a weak yet full agonist of both mouse and human TLR4. E-MPLA, however, revealed markedly different activities as a function of species: in each of the mouse TLR4 cell systems (**Figures 1B, D, F**) it was a robust agonist that stimulated very close to the maximal activity seen with Lipid A, whereas in human TLR4 cell systems (**Figures 1A, C, E**) its peak activity was severely limited and behaved more like a partial than a full agonist. The same limited peak activity in human *versus* mouse cells was observed in reporter cells (**Figures 1A, B**), monocytic cell-lines (**Figures 1C, D**) and primary cells (**Figures 1D, E**) suggesting the pattern is likely to be true *in vivo* in humans as well.

**Figure 2:**
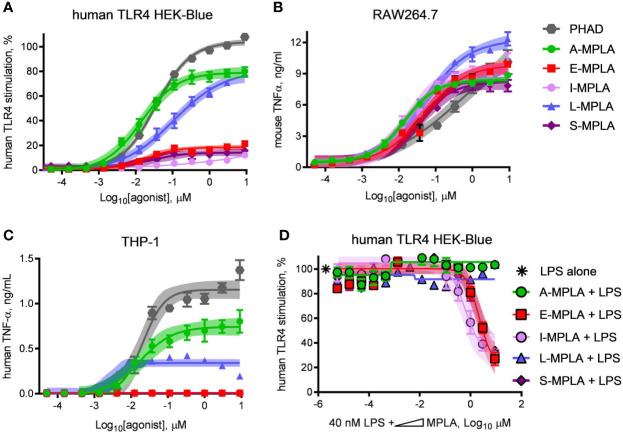
Partial agonist activity of MPLA preparations correlates with presence of TLR4 inhibitors. Research-grade MPLA preparations from five commercial sources were tested for mouse *vs* human TLR4 stimulatory activity. Secretion of alkaline phosphatase by **(A)** human TLR4 HEK-Blue reporter cells was used as a marker of receptor stimulation after exposure to increasing doses of MPLA. TNFα production was used as a marker of TLR4 stimulation by **(B)** RAW264.7 mouse monocytic cells and **(C)** THP-1 human monocytic cells. Inhibition curves were performed with **(D)** human TLR4 HEK-Blue cells exposed to a fixed concentration of LPS alone or in combination with increasing amounts of each MPLA. Symbols and error bars show the average ± SD of **(A, B)** normalized values (100% = top dose plateau of Lipid A from three independent experiments or of **(C, D)** cytokine abundance measured in two independent experiment each performed in triplicate. Shaded regions indicate 99% confidence intervals within which the true population means should occur 99% of the time. MPLA preparations with the lowest dose plateaus for stimulation of human TLR4 [E-, I-, and S-MPLA in **(A)**] were the same as those that inhibited TLR4 stimulation when combined with LPS in **(B)**.

To determine whether partial agonism of human TLR4 was specific to E-MPLA, we tested four other commercial preparations using human TLR4 and mouse TLR4 reporter cells. Two of the preparations, S- and I-MPLA, showed the same limited plateau as E-MPLA in human cells, whereas A-MPLA and L-MPLA were more effective. All preparations, however, were robust agonists of mouse TLR4 signaling as compared to the full (if weak) agonist PHAD (**Figure 2B**). THP-1 response patterns (**Figure 2C**) showed a similar rank order of efficacies amongst the MPLA preparations as did the human TLR4 reporter cells, A-MPLA > L-MPLA >> E-MPLA > S- and I-MPLA, although the responses were even more muted relative to PHAD in THP-1 cell cultures. Given the known heterogeneity of MPL^®^ adjuvant we decided to test the MPLA preparations for evidence of inhibitory activity by serially diluting each of them in a fixed concentration of LPS and testing human TLR4 HEK-Blue cells for responsiveness (**Figure 2D**). The same three MPLA preparations with limited efficacy in THP-1 and human TLR4 reporter cells were also inhibitors of LPS, while the remaining two were not. Hence, only the low efficacy preparations of MPLA demonstrated a hallmark of partial agonists—inhibition of full agonists—but the pattern was not uniformly seen among the different preparations, presumably due to differences in manufacturing.

The various MPLA preparations were evaluated by tandem mass spectrometry to determine which, if any, components were correlated with low dose plateaus. The results of this analysis (**Figure S1**) showed that one component, a tetra-acylated structure designated 4c, was significantly and inversely correlated with the height of the top dose plateau. However, the analysis also revealed the absence of structures de-acylated at the 3-O-position, which highlighted the lack of base hydrolysis used in preparation of the commercial MPLAs. Because clinical MPL^®^ adjuvant is base hydrolyzed and therefore uniformly 3-O-desacylated (10, 16) the partial agonist activity observed in some of the MPLA preparations did not necessarily mean that MPL^®^ adjuvant must also behave as a partial agonist of human TLR4.

### Species-Specific Activity of MPL^®^ Adjuvant

**Figure 3:**
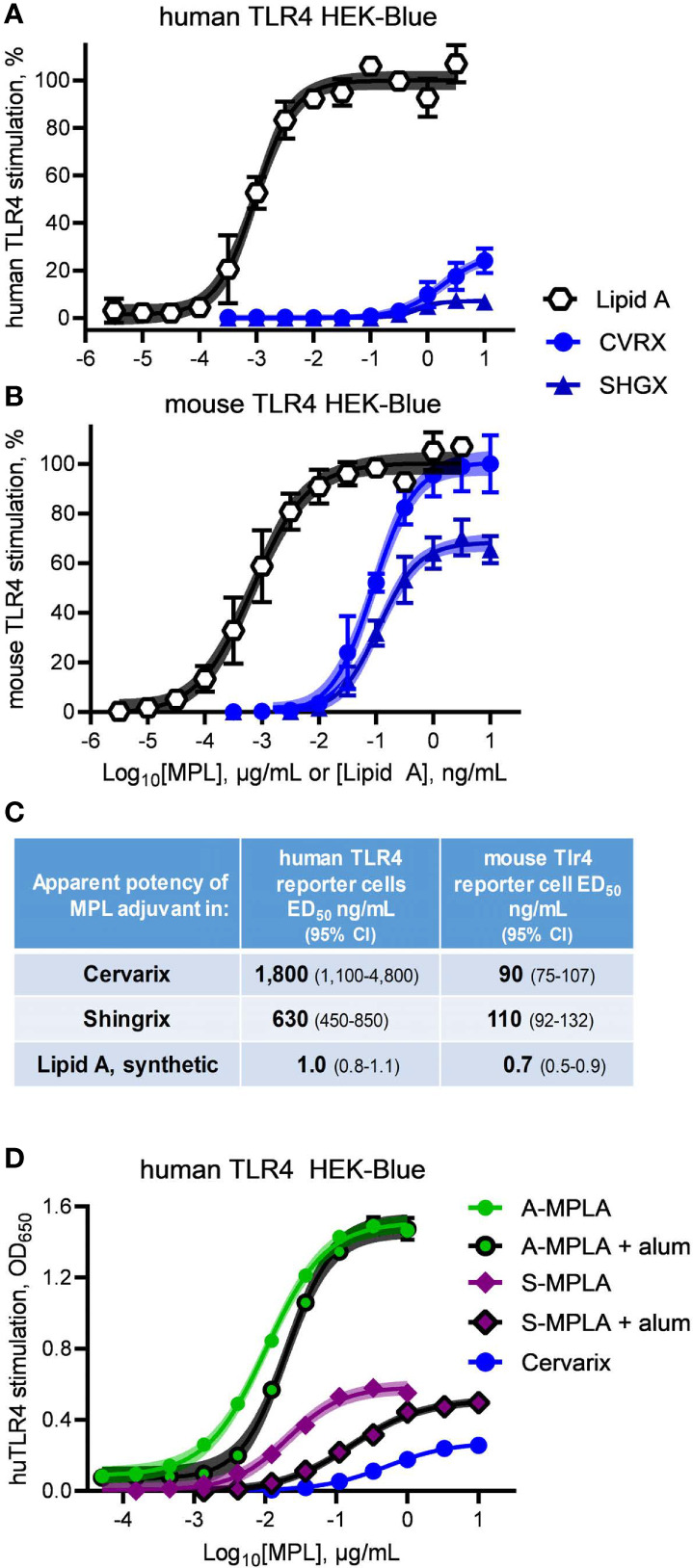
Clinical-grade MPL adjuvant is a partial agonist of human TLR4. Clinical-grade MPL adjuvant^®^ was evaluated for human *vs* mouse TLR4 dose response profiles by exposing **(A)** human TLR4 and **(B)** mTLR4 HEK-Blue reporter cells to Cervarix vaccine (CVRX) or the AS01 adjuvant component of Shingrix vaccine (SHGX) for 24 h. 100% = the top dose plateau for Lipid A-induced secretion of alkaline phosphatase, which was used to normalize TLR4 stimulatory activity between the two reporter cell-lines for a combined total of three independent experiments. **(C)** MPL adjuvant^®^ was 5–16% as potent an agonist of human TLR4 than it was of mouse TLR4 as determined by stimulatory curve fitting to calculate ED50 for the curves shown in **(A, B)**. Low dose plateaus of human TLR4 responses to MPL adjuvant^®^ relative to Lipid A and mouse TLR4 responses, indicate it is a partial agonist of human TLR4. **(D)** human TLR4 HEK-Blue cells were treated with MPLA adsorbed on alum and compared to MPLA alone; alum adsorption did not decrease efficacy of two MPLA preparations. Curve fitting to calculate the top dose plateaus with and without alum produced the following values (with 95% confidence intervals): A-MPLA, 1.51 (1.49–1.53) *vs* A-MPLA + alum, 1.49 (1.47–1.52); S-MPLA, 0.59 (0.57–0.60) *vs* S-MPLA + alum, 0.51 (0.50–0.53). Shaded regions indicate 99% confidence intervals within which the true population means should occur 99% of the time.

We obtained clinical preparations of Shingrix and Cervarix vaccines, which contain MPL^®^ adjuvant in the forms of AS01 and AS04, respectively, and tested their activities as agonists of mouse *versus* human TLR4. Exposure of hTLR4 HEK-Blue reporter cells to serially diluted Cervarix vaccine and contents of the adjuvant vial from Shingrix vaccine resulted in very weak and very low efficacy stimulation of human TLR4 relative to Lipid A (**Figure 3A**). Both clinical preparations were markedly more effective as agonists of mouse TLR4 (**Figure 3B**), with high doses of Cervarix reaching the same dose plateau as Lipid A and high doses of Shingrix adjuvant (*i.e.*, AS01_B_) approaching 70% the efficacy of Lipid A. Relative to Lipid A, the full stimulation benchmark, Cervarix was 27% (95% confidence interval, CI, 23–39%) as effective an agonist of human TLR4 but 100% (CI 97–105%) as effective an agonist of mouse TLR4. Similarly, Shingrix AS01_B_ was 7.2% (CI 6.4–8.0%) *versus* 68% (CI 66–71%) as effective an agonist of human *versus* mouse TLR4, respectively, using Lipid A as the full agonist benchmark. Stimulatory potencies of AS04 and AS01_B_ for mouse TLR4 were 6- to 20-fold higher than for human TLR4 (**Figure 3C**), whereas that of Lipid A was about the same in both. Together, these higher potency and efficacy patterns in **Figure 3B** demonstrate that the low activity in **Figure 3A** was not due to MPL^®^ adjuvant being impaired or unavailable in the HEK-Blue reporter system.

It is formally possible that adsorption of MPL^®^ adjuvant on aluminum hydroxide could be responsible for the limited efficacy of Cervarix in the human TLR4 reporter cell assay, despite the high activity for mouse TLR4 and the fact that the Shingrix AS01_B_ suspension does not contain alum. We did not have access to pure MPL^®^ adjuvant nor was it possible to retrieve it in pure form from the Cervarix suspension so we addressed this possibility by evaluating the effect that alum adsorption had on the efficacies of MPLA preparations. As shown in **Figure 3D**, neither adsorption of high efficacy A-MPLA nor low efficacy S-MPLA on aluminum hydroxide resulted in lower dose plateaus although potencies were reduced, suggesting that alum adsorption reduces the available concentration of MPL^®^ adjuvant but not its maximal activity at high doses. This observation along with the different pharmacological profiles of MPL^®^-adjuvanted vaccines in human *vs* mouse cell systems led us to conclude that research-grade MPLA (**Figures 1** and **2**) and clinical-grade MPL^®^ adjuvant function as partial agonists of TLR4, despite being prepared by distinct manufacturing methods.

**Figure 4:**
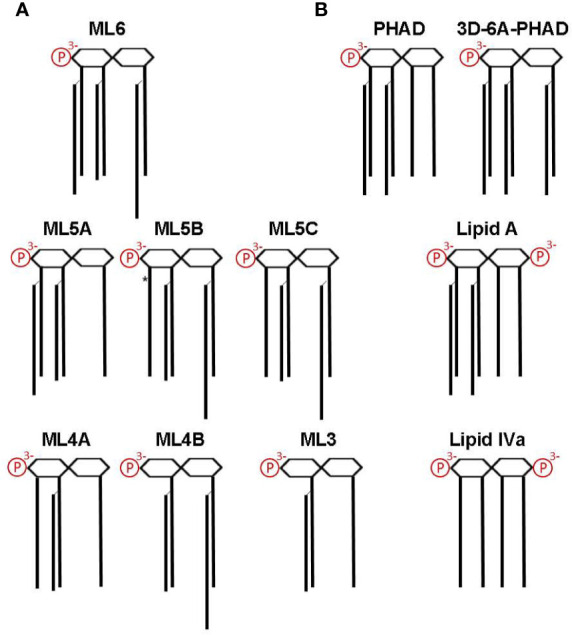
Structures of MPL components and other Lipid A used in this study. Lipid A structures are shown as simplified diagrams representing a *β*-(1→6) di-glucosamine backbone with varying numbers of acyl chains and phosphates; 1- and 4′ phosphorylation sites are depicted on the right and left, respectively. Amino, ether, ester, and hydroxide linkages are omitted. **(A)** The major components of MPL adjuvant^®^ are congeners that differ primarily at the level of acyl chain attachment sites and lengths. All MPL^®^ adjuvant components lack an acyl chain at the 3-position (*i.e.*, are 3-O-deacylated) due to an alkaline hydrolysis processing step. *, denotes location of an unsaturated acyl group that represents the sole difference between ML5B and ML5C. Note that the 2′-position secondary acyl chains are slightly shorter, C12, than the other C14 chains and that the 2-position secondary acyl chain is slightly longer, C16. **(B)** Structures of synthetic Lipid A used in this study. PHAD (phosphorylated hexa-acyl disaccharide) is a synthetic 4′-monophosphorylated lipid A with six C14 acyl chains in the *E. coli* configuration (2:2:1:1); 3D-6A-PHAD is a synthetic 3-O-desacylated variant in the same configuration as ML6 (2:2:0:2) but with uniform acyl chain lengths. Lipid A, synthesized with six acyl chains and 1-, 4′-phosphates, is highly potent agonist of both mouse and human TLR4; Lipid IVa, synthesized with four primary acyl chains and 1-, 4′-phosphates, is an agonist of mouse TLR4 but a potent antagonist of human TLR4.

MPL^®^ adjuvant is a heterogeneous mixture of congeneric lipid A with 4′-phophoryl groups and varying numbers of acyl chains (10, 13, 14, 17–19), as summarized in **Figure 4A**. **Figure 4B** shows other synthetic Lipid A preparations used in our study, including *E. coli* chemotype PHAD and 3D-6A-PHAD, the latter of which is almost identical to the best known component of MPL^®^, hexa-acyl ML6, by virtue of lacking a fatty acid at position 3 of the reducing half of the diglucosamine headgroup. However, 3D-6A-PHAD differs in having uniformly C14-length acyl chains rather than C12 and C16 secondary acyl chains attached at positions 2′ and 2 of the diglucosamine headgroup, respectively. We obtained synthetically prepared versions of most of the major MPL^®^ components and tested them for activity as agonists of mouse *versus* human TLR4.

**Figure 5:**
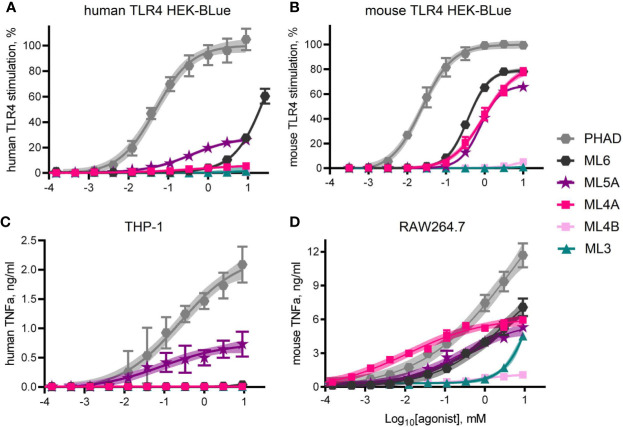
TLR4 stimulatory activity of synthetic MPL adjuvant^®^ components. Synthetic versions of several of the major components of MPL adjuvant^®^ were tested for TLR4 stimulatory activity using HEK-Blue reporter cells that expressed either **(A)** human TLR4 or **(B)** mouse TLR4. Values shown are averages ± SD from three **(A)** or two **(B)** independent experiments normalized by setting 100% = top dose plateau of PHAD in each experiment. Shaded regions indicate 99% confidence intervals within which the true population means should occur 99% of the time. TNFα secretion by **(C)** human THP-1 and **(D)** mouse RAW264.7 cells as measured by ELISA 24 hrs after exposure to agonists in triplicate pooled from two or three independent experiments, respectively, with the exception of ML4A, ML4B and ML3 which were tested in triplicate in one experiment with RAW264.7 cells. Overall, ML5A and ML6 are weaker agonists of human TLR4 than of mouse TLR4, relative to the full agonist PHAD, whereas ML4A is an agonist only of mouse TLR4.

Dose response curves were generated using MPL^®^ components ML6, ML5A, ML5B, ML4A, ML4B, and ML3 to treat human *versus* mouse TLR4 HEK-Blue reporter cells and THP-1 *versus* RAW264.7 cell-lines (**Figure 5**). Curves generated with the full but weak agonist PHAD were used as benchmarks for maximal stimulation of TLR4 in each cell system. The most evident species-specific difference was observed with component ML4A, which was all but inactive when testing human TLR4 HEK-Blue (**Figure 5A**) cells but approached 80% of the efficacy of the full agonist PHAD when testing the mouse counterpart, mouse TLR4 HEK-Blue (**Figure 5B**). A similar pattern was evident when comparing the activity of ML4A in THP-1 and RAW264.7 cultures (**Figures 5C, D**). The other tetra-acylated component we tested, ML4B was largely inactive in all stimulation assays, as was ML3.

ML5A was the most consistently active in human cell systems, even when compared to ML6, whereas both were similarly active in mouse TLR4 HEK-Blue and RAW264.7 cells. Generally speaking, mouse TLR4 seemed unable to distinguish ML4A, ML5A and ML6 from one another while human TLR4 was much more sensitive to acyl chain variation.

### Inhibitory Components in MPL^®^ Adjuvant Mixtures

**Figure 6:**
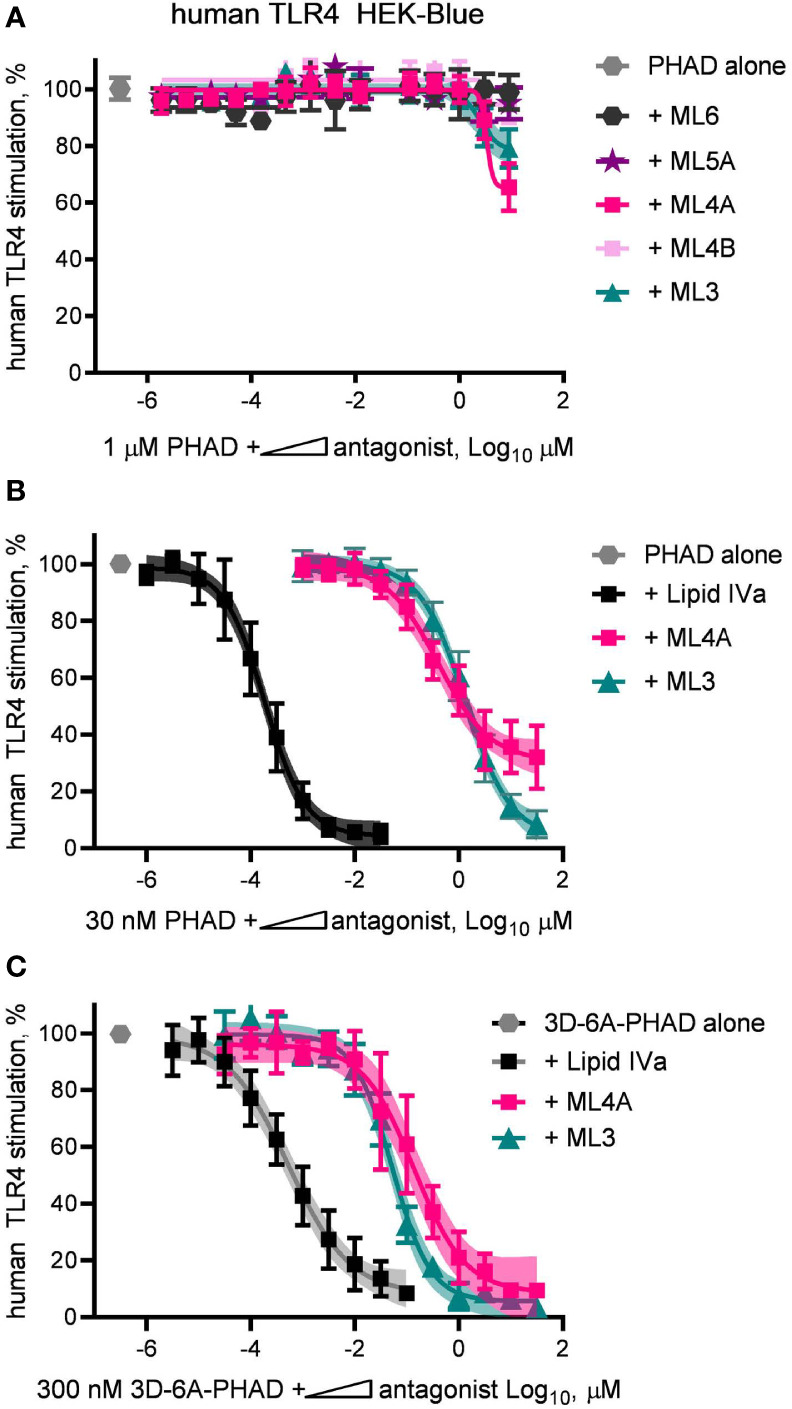
Inhibition of human TLR4 by tetra- and tri-acylated components of MPL adjuvant. Synthetic versions of several major components of MPL adjuvant^®^ were tested for inhibitory activity using human TLR4 HEK-Blue reporter cells. **(A)** MPL adjuvant^®^ components ML3, ML4A, ML4B, ML5A and ML6 were diluted in half-log steps in a mixture containing a fixed concentration of PHAD (1 μM) and added to human TLR4 HEK-Blue reporter cells for 22 h. Values shown are averages ± SD of secreted alkaline phosphatase from two independent experiments, each performed in triplicate and normalized by setting 100% = PHAD alone (1 μM). **(B, C)** MPL adjuvant^®^ components ML3 and ML4A were diluted from a peak dose of 31.6 μM (or a peak dose of 0.03 μM Lipid IVa as antagonist control) in half-log steps in a mixture containing a fixed concentration of PHAD (30 nM) or 3D-6A-PHAD (300 nM), respectively. Values shown are averages ± SD of secreted alkaline phosphatase from two independent experiments normalized by setting 100% = PHAD or 3D-6A-PHAD alone, respectively. Shaded regions indicate 99% confidence intervals within which the true population means should occur 99% of the time. Calculated IC50^PHAD^ values (95% CI) were: ML3, 1.32 μM (1.08–1.67); ML4A, 0.40 μM (0.27–0.62); and Lipid IVa: 0.19 nM (0.15–0.23).

The limited efficacy of some research-grade MPLA preparations were correlated with their ability to inhibit a full agonist of human TLR4 (**Figures 2A, C, D**). This observation led us next to test the synthesized components of MPL^®^ adjuvant for inhibitory activity. To do this, we diluted components ML3, ML4A and B, ML5A and ML6 in a solution containing a fixed concentration of the full agonist PHAD, 1 μM, and plotted the results in the form of an IC50 curve (**Figure 6A**). Of the five components tested, only ML3 and ML4A showed inhibitory potential. These components were tested again but this time using a fixed concentration of PHAD that corresponded to its EC50 in the same cell system, 30 nM (**Figure 6B**). Here, a more complete inhibition curve was generated in which ML3 completed blocked PHAD at a sufficiently high dose. Calculation of its IC50 under these conditions indicated that its inhibitory potency was almost four orders of magnitude weaker than a control antagonist of human TLR4, Lipid IVa. Component ML4A was a slightly more potent inhibitor of PHAD but at high doses the curve appeared to approach a bottom plateau rather than achieving full inhibition. We also tested ML3 and ML4A for inhibitory effects on a PHAD variant, 3D-6A-PHAD, that is similar in structure to that of ML6 in lacking an acyl chain at the 3-position of the diglucosamine headgroup (**Figure 3**) and, as a commercial reagent, is available in more abundant quantities than ML6. We measured the EC50 of 3D-6A-PHAD for stimulation of human TLR4 HEK-Blue reporter cells and used this concentration, 300 nM, as a fixed diluent in which to serially dilute ML3, ML4A, or Lipid IVa (**Figure 6C**). Treatment of human TLR4 reporter cells with these dilution series again showed that all functioned as inhibitors of a reference agonist, 3D-6A-PHAD, that also served as a more accurate surrogate for one of the agonists present in the MPL^®^ adjuvant mixture of congeners.

**Figure 7:**
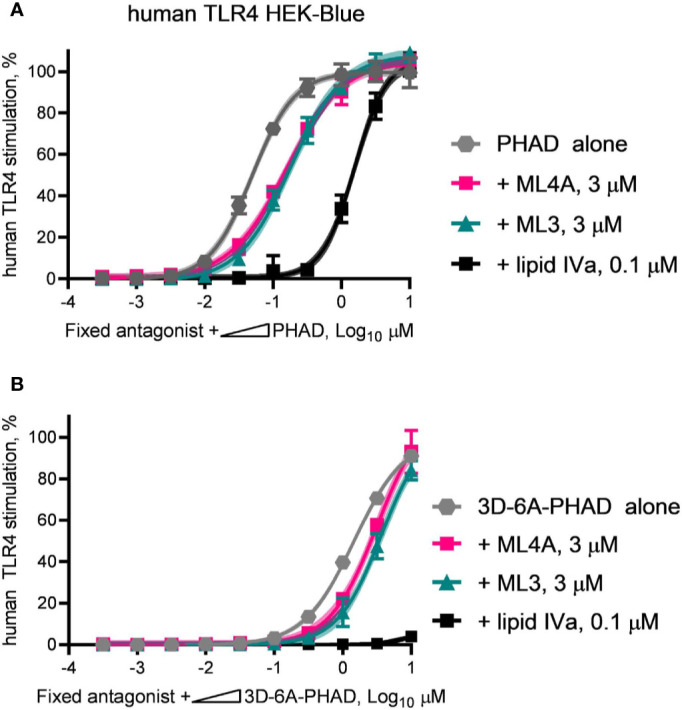
ML3 and ML4A function as competitive antagonists of human TLR4. MPL adjuvant^®^ components ML3 and ML4A were tested for competitive antagonism of human TLR4 using reporter cells. The agonists **(A)** PHAD and **(B)** 3D-6A-PHAD were diluted in half-log steps from a peak concentration of 10 μM in a mixture containing a fixed concentration of ML3 (3 μM), ML4A (3 μM) or Lipid IVa (0.1 μM). Values shown are averages ± SD of secreted alkaline phosphatase from two independent experiments, each performed in duplicate or triplicate and normalized by setting 100% = PHAD or 3D-6A-PHAD alone (3 μM). Shaded regions indicate 99% confidence intervals within which the true population means should occur 99% of the time. Agonist curves in the presence of a fixed amount of ML3 or ML4A reached the same top dose plateaus as for agonist alone indicating both are competitive antagonists of human TLR4.

We next evaluated the antagonist type, competitive or non-competitive, of the ML3 and ML4A components by using them as fixed concentration diluents in dilution series of PHAD (**Figure 7A**) or 3D-6A-PHAD (**Figure 7B**). Both ML3 and ML4A shifted the potency curves of the agonists without preventing either PHAD or 3D-6A-PHAD from fully stimulating the human TLR4 reporter cells as the agonist concentrations rose, the hallmark of a competitive antagonist that can be out-competed by sufficiently high concentrations of agonist. The Lipid IVa control produced a competitive antagonist curve when mixed with PHAD, as expected, but its inhibitory effect could not be overcome by the concentrations we used when testing 3D-6A-PHAD, highlighting the weakness of the 3-O-deacylated derivative as an agonist of human TLR4. Overall, the patterns observed in **Figures 6** and **7** showed that two of the major MPL^®^ components, tri-acylated ML3 and tetra-acylated ML4A, were competitive antagonists of human TLR4.

### Mixed Agonist–Antagonist Preparations

**Figure 8:**
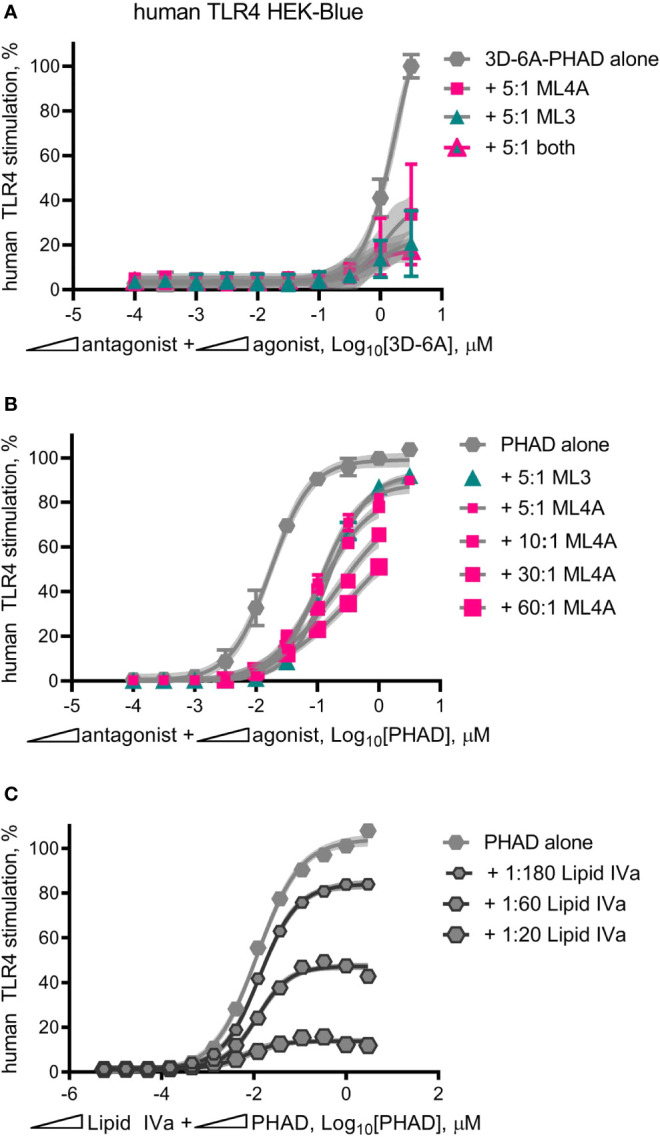
Mixed agonist–antagonist dose profiles replicate the low dose plateau of MPL adjuvant^®^. Dose curve profiles of agonist-antagonist mixtures at fixed ratios were generated using human TLR4 reporter cells to determine whether the suppressed dose plateau of MPL adjuvant^®^ could be recapitulated with synthetic components. **(A)** 3D-6A-PHAD, as a surrogate for ML6, was mixed with either ML3, ML4A or both at a ratio of 5:1 antagonist:agonist and diluted in half-log steps from peak concentrations of [3.16 μM 3D-6A-PHAD + 15.8 μM antagonist]. Values ± SD shown are of secreted alkaline phosphatase from three independent experiments performed in duplicate and normalized by setting 100% = max value for 3D-6A-PHAD alone. **(B)** PHAD was mixed with ML4A at ratios of 5:1, 10:1, 30:1 or 60:1 antagonist:agonist and diluted in half-log steps from peak concentrations of either 3.16 μM (5:1) or 1 μM (10:1, 30:1, 60:1) PHAD; two experiments were normalized by setting 100% = top dose plateau for PHAD alone. **(C)** PHAD was mixed with Lipid IVa at ratios of 1:20, 1:60 or 1:180 antagonist:agonist and diluted in half-log steps from a peak concentration of 3.16 μM PHAD; Values ± SD are from three independent experiments, each in triplicate, normalized by setting 100% = top dose plateau for PHAD alone. Shaded regions indicate 99% confidence intervals within which the true population means should occur 99% of the time. Mixed agonist:antagonist preparations containing synthetic PHAD and Lipid IVa generated the largest dose range across which a suppressed plateau of human TLR4 stimulation could be maintained.

MPL^®^ adjuvant, in the form of intact Cervarix vaccine or the adjuvant vial of Shingrix, appeared to function as a partial agonist of human TLR4, with a limited dose plateau even at high doses (**Figures 3A, D**). However it was possible this pattern was attributable to the other compounds present, such as viral antigen in Cervarix or the saponin QS-21 in AS01_B_. To determine if we could re-capitulate partial agonism of human TLR4 without these other compounds, we mixed 3D-6A-PHAD with either ML3, ML4A or ML3+ML4A at a 5:1 ratio, prepared serial dilutions at this fixed ratio and compared the TLR4 stimulatory activities of the mixtures to that of 3D-6A-PHAD alone (**Figure 8A**). Although the weakness of the 3D-6A-PHAD agonist made it difficult to achieve a clear dose plateau, the addition of ML3 and ML4A appeared to limit its activity in a manner that was reminiscent of the curves observed for Cervarix or the Shingrix AS01_B_ adjuvant.

Repetition of this experiment using the comparatively stronger agonist PHAD (**Figure 8B**) generated curves in which ML3 and ML4A very modestly reduced TLR4 stimulatory activity without establishing a flattened dose plateau, even when ML4A and PHAD were mixed at fixed ratios of 30:1 and 60:1 antagonist:agonist. To determine whether or not this failure to convert PHAD from a full agonist to a partial agonist (in the form of a mixed agonist-antagonist formulation) was due to the weakness of ML3 and ML4A inhibitory effects we then performed mixed agonist-antagonist assays using the potent antagonist Lipid IVa in place of ML3 and ML4A (**Figure 8C**). In this case, the PHAD dose response curve was trending to a flattened curve with as little as one part Lipid IVa to 180 parts PHAD and was clearly flatted when using lower ratios of agonist, 1:60 or 1:20 antagonist:agonist. Collectively, the patterns observed in **Figures 8A–C** indicated that a full agonist of TLR4 could be made to function as if it were a partial agonist by adding an antagonist at a fixed ratio, provided the antagonist was of sufficient inhibitory potency relative to the agonist being tested. We concluded, therefore, that the partial agonism of human TLR4 by MPL^®^ adjuvant preparations AS01B and AS04 could be explained by the presence of a mixture of agonists and antagonists of the receptor.

## Discussion

We identified two antagonists of human TLR4 in clinical-grade MPL^®^ adjuvant, components ML3 and ML4A which are tri- and tetra-acylated, respectively. We propose their presence is an additional mechanism by which chemical processing detoxifies *S. enterica* serovar Minnesota Re595 LPS for therapeutic use. The structural determinants of therapeutic detoxification that have been documented in the literature include i) loss of inner core saccharides (30), ii) loss of the 1-phosphate (14)., iii) loss of the 3-O-acyl chain (10) and now, we suggest, iv) the presence of antagonists that prevent full occupancy of TLR4 such that the overall mixture behaves as a partial agonist.

Our finding that component ML4A is an agonist of mouse TLR4 (**Figures 5B, D**) but an antagonist of human TLR4 (**Figure 6**) is consistent with the behavior of another tetra-acylated structure, Lipid IVa. The known presence of tetra-acylated congeners in MPL^®^ adjuvant was in fact one of the rationales for our decision to evaluate the species specificity effects of both the clinical-grade and the research-grade versions of the adjuvant mixture. Product information provided by the manufacturers of the various MPLA preparations indicates that none of the sources we used, or could find, included the alkaline hydrolysis step described by Myers and colleagues (10, 16). Omission of this step, which specifically releases the 3-O-acyl chain of Lipid A, means that the structure of the tetra-acylated component that appears to be present in some of the MPLA preparations we tested (E-, I-, and S-MPLA; **Figure 2D**) is not likely to be identical to ML4A. A likely candidate is a monophosphorylated version of Lipid IVa that would retain a 3-O-acyl chain in a 1:1:1:1 configuration (four primary acyl chains). Confirmation that this is true would require detailed analysis of the structures present in the various MPLA preparations.

A somewhat more surprising finding than the presence of a tetra-acylated antagonist of human TLR4 in MPL^®^ adjuvant is the existence of a tri-acylated antagonist, component ML3. A search of the literature revealed that this structure**–**activity relationship is not unprecedented because synthetic compounds similar in structure to ML3 but with two phosphates, or one phosphate and non-physiological R groups placed at the 1-position, were reported to inhibit human TLR4 several years ago (31). Although the abundance of ML3 in MPL^®^ adjuvant preparations is unclear because studies of MPL^®^ composition were generally focused on analysis of structures with four or more acyl chains (10, 13, 14, 17, 20), its inhibitory activity likely overlaps with that of the more abundant ML4A structure to form a mixture of TLR4 agonists and antagonists whose combined activity is typical of partial agonism, ie, partial stimulation of a signaling system through non-saturating engagement of its upstream receptors or downstream effector molecules. In the case of ML3 and ML4A, it is probable that upstream receptor occupancy is affected due to their behavior as competitive antagonists in the potency shift assays we performed (**Figure 7**).

We also report a technique whereby the partial agonism of human TLR4 by MPL^®^ adjuvant can be replicated with a far simpler mixture of components. In the example provided (**Figure 8C**), mixtures of the full agonist PHAD with the antagonist Lipid IVa generated dose response curves in the human TLR4 HEK-Blue system whose top dose plateau could be manipulated by adjusting and then fixing the relative proportions of the two compounds. This ‘antagonist spiking’ approach has the advantages of simplifying the number of components to be monitored in production of vaccine lots, relative to the half-dozen present in MPL^®^ adjuvant whose proportions are sensitive to minor differences in temperature and acid and alkaline hydrolysis conditions (10) and reducing the amount of material needed to achieve a desired threshold of TLR4 receptor occupancy and stimulation (**Figure 8C**).

It remains to be determined whether or not partial agonism of TLR4 by a mixed agonist-antagonist preparation is actually beneficial. Consideration of the frequency of reactogenic side effects reported in clinical trials of vaccines containing MPL^®^ adjuvant, however, suggest that it may be critically important at the level of maintaining tolerability in healthy human subjects. A review of clinical trial data from head-to-head comparisons of vaccines that differed solely or primarily in having MPL^®^ adjuvant or not (*i.e.*, alum alone *versus* AS04 formulations) revealed that intramuscular immunizations produced painful inflammatory reactions in nearly 90% of study participants when the vaccine included MPL^®^ adjuvant *versus* 40–60% when it did not (32). This pattern was surprisingly uniform across differing demographics that included girls age 9–14 (33), women aged 18–45 (34), or men and women aged 18–45 (35). Removal of adjuvant components such as ML3 and ML4A that moderate the extent to which TLR4 is activated could potentially exacerbate painful injection sites and other indices of local inflammatory reactions. Hence, it is possible that the current composition of MPL^®^ adjuvant is approaching the asymptote of tolerability that influences when a vaccine is considered to be safe such that increases in its TLR4 stimulatory function could be counterproductive.

Interindividual human variability of TLR4 responses further complicates the safe use of an endotoxin-derived material in vaccines. Cervarix and Shingrix injections deliver MPL^®^ adjuvant at an initial concentration of 100 μg/ml, which could theoretically be decreased if endogenous antagonists were somehow removed. A low dose of a full TLR4 agonist presumably could be used to achieve therapeutic effects below the threshold for endotoxic effects in some individuals, but achieving the same outcome consistently in large numbers of diverse people, as is the case with prophylactic immunization seems problematic. This is because some individuals appear to be more sensitive to endotoxin signaling than others. For example, in a study of 102 healthy blood donors whose cells were exposed to 10 ng/ml LPS for 6 h the amount of TNFα produced varied by three orders of magnitude (36). This enormous range of sensitivities suggests that methods by which TLR4 receptor occupancy is minimized, fortuitously or by design, could be beneficial by putting an upper limit on the extent to which the signaling system can be activated. Our study thus identifies mixed agonist-antagonist activity as an additional mechanism by which MPL^®^ adjuvant is detoxified to render it safe for widespread use in the vast populations of individuals in need of immune protection from infectious disease.

## Data Availability Statement

All datasets presented in this study are included in the article/**Supplementary Material**.

## Ethics Statement

The studies involving human participants were reviewed and approved by University of Louisville Institutional Review Board. The patients/participants provided their written informed consent to participate in this study. The use of mice as a source of bone marrow was reviewed and approved by Institutional Animal Use and Care Committee.

## Author Contributions

TM, CC, JE, and HB-L conceived of the project. Y-QW wrote sections of the manuscript with finalization of it by TM and CC. Y-QW, CC, and TM performed experiments as described in the text. All authors contributed to the article and approved the submitted version.

## Funding

Research reported in this publication was supported by the National Institute of Allergy and Infectious Diseases of the National Institutes of Health under Award Number R01AI127970. The content is solely the responsibility of the authors and does not necessarily represent the official views of the National Institutes of Health. Additional funding was provided by the Barnstable-Brown Foundation and the Commonwealth of Kentucky Research Challenge Trust Fund.

## Conflict of Interest

The authors declare that the research was conducted in the absence of any commercial or financial relationships that could be construed as a potential conflict of interest.
