# Supplementary figures and images for: MPL Adjuvant Contains Competitive Antagonists of Human TLR4
Source: Front Immunol. 2020 Oct 16;11:577823. doi: 10.3389/fimmu.2020.577823 (PMC7596181; doi:10.3389/fimmu.2020.577823)

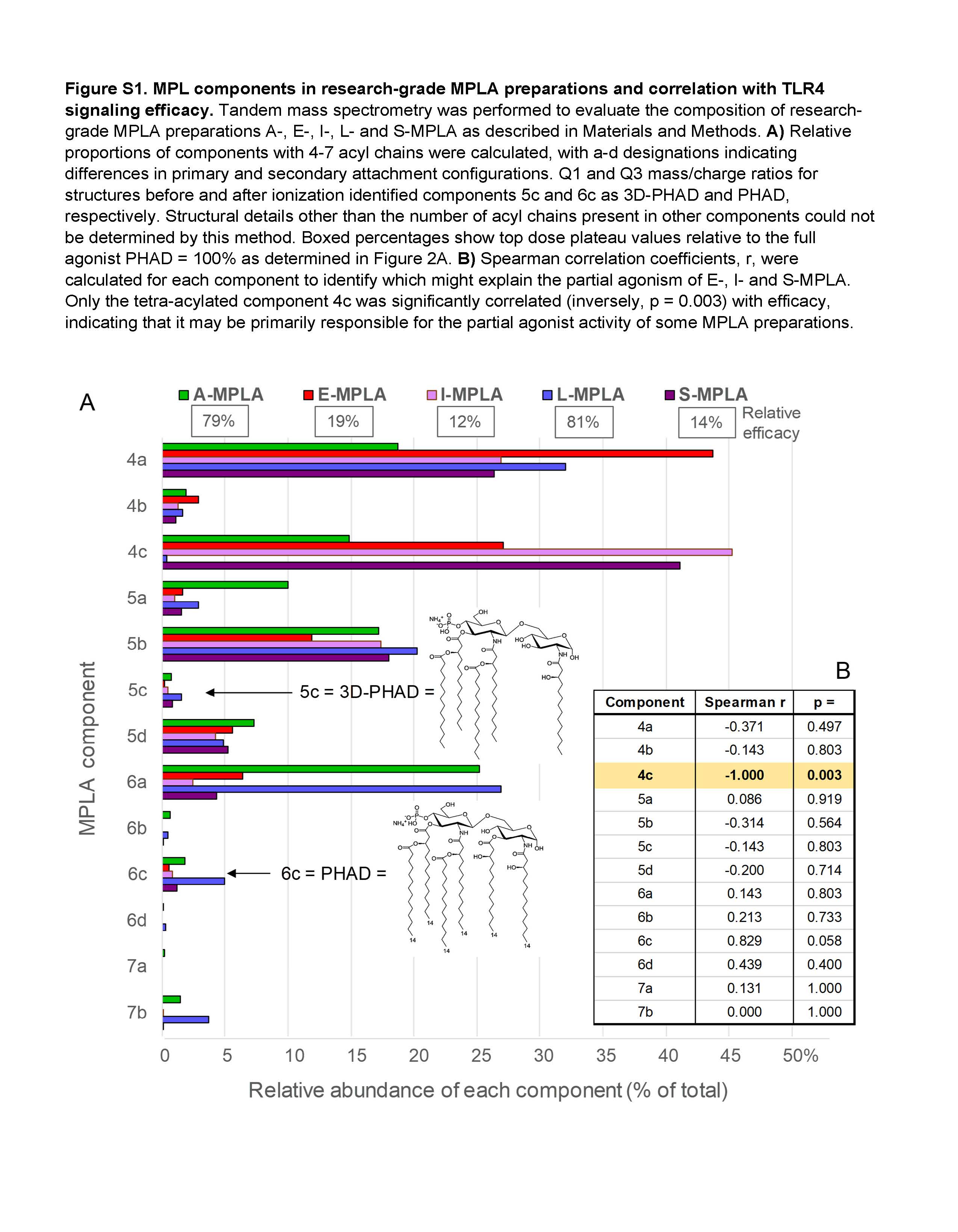

Supplement: Supplementary file 1 [file Image_1.jpeg]
